# A Prospective Study to Assess Patients' Understanding and Retention of Postoperative Instructions following a Fixed Partial Denture, Using Different Information Delivery Techniques

**DOI:** 10.1055/s-0042-1750774

**Published:** 2022-09-05

**Authors:** Hafiz Adawi, Saurabh Jain, Aeshah H. Hakami, Naseem A. Mtwam, Almaha Y. A. Koriri, Abdulaziz A. A. Adawi

**Affiliations:** 1 Department of Prosthetic Dental Sciences, College of Dentistry, Jazan University, Jazan, Saudi Arabia; 2 Dental Intern, College of Dentistry, Jazan University, Jazan, Saudi Arabia; 3 Armed Forces Hospital, Jazan, Saudi Arabia

**Keywords:** at-home maintenance instructions, fixed partial denture, instruction delivering modes, patient education, patient understanding

## Abstract

**Objective** This study was aimed to evaluate the effect of using different modes of at-home maintenance information delivery on patients' understanding and the level of information retention.

**Materials and Methods** Sixty patients were asked to answer a questionnaire while undergoing mouth preparations for receiving a fixed partial denture. The questionnaire includes questions related to at-home maintenance procedures and recall visits. After finishing the first questionnaire, these patients were randomly assigned into three groups, and each group was given education about at-home maintenance procedures and recall visits by different means. Group 1 participants were educated by giving live demonstrations. Group 2 participants were shown a prerecorded video, whereas group 3 participants were given written instructions. All Participants were recalled after 1 week of the last visit and were asked to fill out the same questionnaire.

**Statistical Ananlysis** Collected data were tabulated in a Microsoft Excel Spreadsheet and were analyzed using the Chi-square test, one-way ANOVA, paired t-test, and post hoc Bonferroni test. A *p* -Value < 0.05 was regarded as statistically significant for all the analyses.

**Results** Group 1 showed greatest information retention as compared with groups 2 and 3 ( *p* = 0.045). There was significant difference in the knowledge of group 1 as compared with groups 2 ( *p* = 0.020) and 3 ( *p* = 0.048).

**Conclusion** The mode of delivering postoperative instructions after fixed partial denture treatment does have an effect on the patient understanding level and information retention. Patients who were given live demonstrations showed the best results compared with video recordings and written leaflets.

## Introduction

For any treatment to be a long-term success, both patient and doctor play an equally important role. Treatment by fixed partial dentures (FPD) prosthesis is no exception. Tooth-supported FPD are considered the standard mode for rehabilitating a patient with single or multiple missing teeth. 1 2 3 Following completion of FPD treatment, detailed instructions regarding at-home maintenance and recall protocol are provided to the patient within a short period of time. This can lead to confusion and inability to retain this information which adversely affects the home maintenance and longevity of the prosthesis.

Previous studies show that one of the commonest causes of failure of these FPDs is plaque accumulation 4 5 6 which can be the primary cause for caries and periodontal disease. This can be attributed to poor at-home maintenance and failure to visit the dentist for regular check-ups and maintenance. De Backer et al found that biological reasons accounted for 69% of failures of the fixed prosthesis (caries being the most common reason for failure, followed by periodontal problems). 7

Postoperative dental care, including oral hygiene maintenance and follow-up dental visits, is key to the long-term success of these restorations. The literature mentions the clinical practice guidelines for dentist, including patient recall, at-home maintenance, and professional maintenance. 8 For a patient to follow these maintenance protocols, the dentist must provide all the instructions through an understandable mode of delivery. The patient's understanding and retention of these instructions play a vital role in the long-term survival of the prosthesis. The standard modes of providing instructions to patients include verbal communication, live demonstrations, educational videos, and written leaflets. 9 10 11 12 Various studies in different fields of health sciences have found that patients find it difficult to understand and retain instructions given verbally. 9 10 11 The use of audio-visual aids for providing oral health education has improved the oral health knowledge of the target groups. 13 14 15

There is a paucity of studies in the literature that focus on the mode of delivering maintenance instructions on patient's understanding and information retention after FPD. This study aims to evaluate the effect of using different modes of at-home maintenance information delivery (demonstration, written, and video) on patient's understanding and the level of information retention. The null hypothesis was that different modes of at-home maintenance information, delivery after FPD cementation, has no effect on patient's understanding and the level of information retention.

## Materials and Methods

### Study Design, Population, and Sample Size Calculation

This randomized controlled study was approved by the institutional review board of College of Dentistry, Jazan University (no: REC41/1–018, date: November 18, 2019). The study was conducted to evaluate the effect of using different information delivery techniques on patient's understanding and level of information retention. The patients involved were informed about the purpose of the study and written consent was taken from the patients willing to participate in the study. Participants include adult patients visiting the comprehensive care clinics of the college of dentistry (from September 2020 to February 2021) to get rehabilitated by fixed partial denture prosthesis. Inclusion criteria were as follows: participants are Saudi citizens, aged between 18 and 60 years and should be fluent in Arabic language and are undergoing rehabilitation by tooth-supported fixed partial denture prosthesis. Exclusion criteria were age less than 18 years and more than 60 years, non-Saudi, and any medical condition that does not permit the understanding of the procedure (patient with mental disability or neurological problems). Pilot study was conducted on 10 patients, and they were not included in the study. The sample size was estimated using G*Power software (version 3.0). A sample size of 60 (20 in each group) was found to be sufficient for an *α* of 0.05, power of 80%, and effect size of 0.80. There are no conflicts of interest to be reported, and it was a self-funded research.

### Study Materials

An educational video comprising all postoperative instructions for home care maintenance (in Arabic) was filmed. Postoperative instructions for home care maintenance were written in the Arabic language. Both video and written instructions were reviewed and approved by all researchers and five specialists from the department of prosthetic dental sciences.

### Assessment Tool and Method of Administration

A self-designed questionnaire comprising 15 closed-ended questions related to the awareness and understanding of postoperative instructions following fixed prosthodontic treatment was used. Additional information related to age, marital status, number of children, education level, occupation, and monthly income was also inquired. The questionnaire was first subjected to pilot testing to evaluate face and content validity. The questionnaire was given to three specialists from the Department of Prosthetic Dental Sciences (who have good knowledge and understanding of postoperative instructions following fixed prosthodontic treatment) for content validity. For evaluating face validity, the questionnaire was given to 10 patients (who are undergoing fixed prosthodontic treatment in the college but are not included in the study) through interviews, and level of understanding of questions was discussed. Questionnaire was rated as relevant and easy to comprehend. Test–retest reliability gave a Cronbach's *α* value of 0.81( *p* < 0.05) when the questionnaire was retested after 7 days.

Sixty patients who were found eligible for this study were asked to answer the first questionnaire while undergoing mouth preparations for receiving an FPD. The questionnaire includes questions related to at-home maintenance procedures and recall visits. After finishing the first questionnaire, these patients were randomly assigned into three groups of 20 each, and each group was given education about at-home maintenance procedures and recall visits by different means. Group 1 participants were educated by giving live demonstrations by pretrained dentists (A.H.H. and N.A.M.). Demonstrations were similar to the prerecorded videos. Group-2 participants were shown a prerecorded video, whereas group-3 participants were given written instructions in the form of a leaflet. All participants were recalled after 1 week of the last visit and were asked to fill the same questionnaire again to evaluate the patients' information retention regarding the at-home maintenance procedure and recall visits, using different information delivery techniques. The researchers (A.Y.A.K. and H.A.) responsible for managing patients during questionnaire form fill-up were blinded regarding participant's assigned group.

### Data Analysis

Descriptive data analysis was performed to synopsize the information. There was no missing data. Collected data were tabulated in a Microsoft Excel Spreadsheet (Microsoft Inc., Redmond, Washington, United States), and statistical analysis was performed using SPSS version 24.0 (IBM Corp. Released 2016; IBM SPSS Statistics for Windows, Version 24.0. Armonk, New York, United States: IBM Corp.). Data were analyzed using the Chi-square test, one-way analysis of variance (ANOVA), paired *t* -test, and post hoc Bonferroni's test. A *p* -value of <0.05 was regarded as statistically significant for all the analyses.

## Results

The present prospective study was conducted on 60 patients attending comprehensive care clinics at college of dentistry. Descriptive statistics of the study population is presented in Table 1 . The study participants were in the age range of 18 to 60 years, with 45% in the age group of 18 to 34 years and 55% in 35 to 60 years. More than half of the participants were married (68.3%), 21.7% were single and only 10% were separated. Most of the participants had four to six children, and majority of them received elementary (38.3%) and high school education (35%). Most of the participants were working in the private sector (75%). Also, 41.7% of the participants have a monthly income of <3,000 Saudi Riyals, 26.7% had a monthly income of 3,000 to 7,500 Saudi Riyals and 18.3% reported a monthly income of more than 10,000 Saudi riyals.

**Table 1:** Descriptive statistics of the study population

Variables		Groups			Total	*p* -Value
		Group 1 *n* (%)	Group 2 *n* (%)	Group 3 *n* (%)		
Age groups (y)	18–34	8 (40)	10 (50)	9 (45)	27 (45)	0.817
	35–60	12 (60)	10 (50)	11 (55)	33 (55)	
Marital status	Married	15 (75)	10 (50)	16 (80)	41 (68.3)	0.033
	Separated	2 (10)	1 (5)	3 (15)	6 (10)	
	Single	3 (15)	9 (45)	1 (5)	13 (21.7)	
Number of children	None	4 (20)	10 (50)	2 (10)	16 (26.7)	0.061
	1–3	6 (30)	2 (10)	8 (40)	16 (26.7)	
	4–6	8 (40)	4 (20)	7 (35)	19 (31.7)	
	>7	2 (10)	4 (20)	3 (15)	9 (15)	
Education level	Illiterate	4 (20)	2 (10)	0	6 (10)	0.226
	Elementary	7 (35)	9 (45)	7 (35)	23 (38.3)	
	High school	5 (25)	6 (30)	10 (50)	21 (35)	
	intermediate	2 (10)	3 (15)	3 (15)	8 (13.3)	
	Graduate/postgraduate	2 (10)	0	0	2 (3.3)	
Occupation	Government employee	2 (10)	1 (5)	0	3 (5)	0.544
	Laborer	0	0	2 (10)	2 (3.3)	
	Private sector	14 (70)	15 (75)	16 (80)	45 (75)	
	Student	2 (10)	2 (10)	1 (5)	5 (8.3)	
	Unemployed	2 (10)	2 (10)	1 (5)	5 (8.3)	
Monthly income (Riyals)	<3,000	11 (55)	7 (35)	7 (35)	25 (41.7)	0.394
	3,000– < 7,500	3 (15)	5 (25)	8 (40)	16 (26.7)	
	7,500– < 10,000	3 (15)	2 (10)	3 (15)	8 (13.3)	
	>10,000	3 (15)	6 (30)	2 (10)	11 (18.3)	

Note: Chi-square test applied, *p* -Value significant at *p* < 0.05.

Data on, information dissemination knowledge before intervention (preintervention) and information retention among the study population post–information dissemination is presented in Table 2 . The questionnaire was given to all the patients before educating regarding at-home maintenance which included questions about the at-home maintenance procedures and recall visits: oral hygiene habits, number of cleaning per day, type of toothpaste, mouth wash, maintaining proper hygiene underneath, between the fixed dental prostheses, dental cleaning aids, and duration of recall visits. Group 3 demonstrated more (25%) recruits having three or more recall visits as compared with groups 1 and 2, and this difference was statistically significant ( *p* = 0.023). Most of the participants brushed their teeth, and the most common cleaning tool used was toothbrush in all three groups. Participants in group 1 usually brushed their teeth only once with regular toothpaste, while those in groups 2 and 3 brushed twice or more with fluoridated toothpaste. Mouthwash was used more in group 3 as compared with groups 1 and 2 ( *p* = 0.001). Most of the people rinsed their mouths with water only. Most of them reported that they had no knowledge about cleaning the prosthesis.

**Table 2:** Information dissemination knowledge before intervention (preintervention) and information retention among study population post–information dissemination

Question		Preintervention				Postintervention			
		Group 1 *n* (%)	Group 2 *n* (%)	Group 3 *n* (%)	*p* -Value for intergroup comparison	Group 1 *n* (%)	Group 2 *n* (%)	Group 3 *n* (%)	*p* -Value for intergroup comparison
The number of recall visits to the dentist per year after treatment	0	15 (75)	16 (80)	7 (35)	0.026	0	10 (50)	0	0.122
	1	3 (15)	0	5 (25)		9 (45)	3 (15)	11 (55)	
	2	2 (10)	2 (10)	3 (15)		7 (35)	6 (30)	3 (15)	
	3 or more	0	2 (10)	5 (25)		4 (20)	1 (5)	6 (30)	
Do you brush your teeth daily?	No	2 (10)	3 (15)	1 (5)	0.0001	0	1 (5)	1 (5)	0.0001
	Yes	18 (90)	17 (85)	19 (95)		20 (100)	19 (95)	19 (95)	
What kind of cleaning tool do you use?	Electric brush	0	0	1 (5)	0.654	0	0	1 (5)	0.007
	Miswak	1 (5)	0	0		0	0	2 (10)	
	Toothbrush	15 (75)	15 (75)	12 (60)		2 (10)	5 (25)	10 (50)	
	Combination of above	4 (20)	5 (25)	7 (35)		18 (90)	15 (75)	7 (35)	
How many times do you brush your teeth per day	1	12 (60)	6 (30)	6 (30)	0.083	2 (10)	0	2 (10)	0.474
	2	6 (30)	11 (55)	7 (35)		14 (70)	15 (75)	11 (55)	
	3 or more	2 (10)	3 (15)	7 (35)		4 (20)	5 (25)	7 (35)	
What kind of toothpaste are you using?	F toothpaste	6 (30)	15 (75)	12 (60)	0.048	16 (80)	16 (80)	9 (45)	0.075
	None	1 (5)	0	0		0	0	0	
	Regular toothpaste	13 (65)	5 (25)	8 (40)		4 (20)	4 (20)	11 (55)	
Do you use mouthwash?	No	16 (80)	14 (70)	9 (45)	0.0001	5 (25)	7 (35)	7 (35)	0.0001
	Yes	4 (20)	6 (30)	11 (55)		15 (75)	13 (65)	13 (65)	
What type of mouthwash you are using?	Chlorhexidine	2 (10)	4 (20)	2 (10)	0.030	3 (15)	3 (15)	1 (5)	0.222
	Regular	1 (5)	2 (10)	2 (10)		7 (35)	8 (40)	2 (10)	
	Water rinse	14 (70)	12 (60)	5 (25)		3 (15)	5 (25)	6 (30)	
	Salt water	0	1 (5)	0		1 (5)	2 (10)	0	
	Combination	3 (15)	1 (5)	11 (55)		6 (30)	2 (10)	11 (55)	
Are you aware that cleaning should be done underneath the prosthesis and in areas between teeth and prosthesis?	No	18 (90)	19 (95)	16 (80)	0.0001	0	3 (15)	12 (60)	0.0001
	Yes	2 (10)	1 (5)	4 (20)		20 (100)	17 (85)	8 (40)	

Note: Chi-square test applied, *p* -Value significant at *p* < 0.05.

The study participants were educated about at-home maintenance procedures, following which the questionnaire was again administered to them to assess their information retention and behavior postintervention. Postinformation, the knowledge of the number of recall visits in all the three groups increased. All the group-1 participants started using a toothbrush as compared with 95% in groups 2 and 3. Almost all the participants in group 1 used toothbrush alone or in combination with other aids and brushed at least once daily. Also, 80% of the participants in groups 1 and 2 used fluoridated toothpaste and 20% used regular toothpaste. Further, 75% started using mouthwash in group 1 compared with 65% in groups 2 and 3. All participants in group 1 had knowledge about how to clean their prosthesis as compared with 80% in group 2 and 40% in group 3. Significant differences in the distribution of participants in three groups could be seen with respect to brushing teeth, use of cleaning aids, mouthwash, and cleaning prosthesis.

Knowledge scores among the three different groups before and after intervention are presented in Table 3 . When the knowledge scores among the three groups were compared before and after giving information regarding at-home maintenance procedures, it was found that group 1 showed the greatest information retention as compared with groups 2 and 3 ( *p* = 0.045). There was a significant difference in the knowledge of group 1 as compared with group 2 ( *p* = 0.020) and group 3 ( *p* = 0.048).

**Table 3:** Knowledge scores among the three different groups before and after intervention

Modes of intervention	Before	After	*p* -Value b
Demonstration (group 1)	55.0 ± 18.8	79.0 ± 18.8	0.0001
Video (group 2)	50.6 ± 19.2	73.7 ± 16.2	0.0001
Written (group 3)	49.3 ± 15.8	74.5 ± 13.8	0.0001
*p* -Value a	0.003	0.045	
*p* -Value (group 1 vs. group 2) c	0.317	0.020	
*p* -Value (group 1 vs. group 3) c	0.002	0.048	
*p* -Value (group 2 vs. group 3) c	0.160	1.00	

a One-way analysis of variance (ANOVA) applied.

b Paired *t* -test applied.

c Post hoc Bonferroni's test applied, *p* -Value significant at *p* < 0.05.

## Discussion

This study analyzed the effect of different modes of delivering maintenance information, after FPD cementation, on patient understanding and information retention. There was a significant difference in patient understanding and level of information retention by varying the mode of delivering the information. Therefore, the null hypothesis can be rejected; however, the extent of understanding and level of information retention varied according to the mode of delivering information to the patient.

Proper at-home maintenance of FPD is essential to increase the longevity of the prosthesis. 16 In this study, posttreatment instructions were delivered to patients in three different ways, that is, giving live demonstrations, showing prerecorded videos, and giving written instructions in the form of a leaflet. It was found that the group of patients who were given live demonstrations showed the greatest information retention compared with other groups. This difference was statistically significant. There was no significant difference in information retention when information was delivered by prerecorded video or written leaflets.

Rossi et al assessed the usefulness of various modes of patient education related to threats, advantages, and choices of treatment before authorizing a consent form. They found that the video group performed better than the verbal group (by 40.1%) when compared with determine comprehension and retention. 17 A similar study was conducted by Agre et al to evaluate the effectiveness of a videotaped presentation compared with an in-person discussion in conveying information to patients undergoing colonoscopy. They concluded that video groups had significantly better scores than those in the discussion-only group. 18 Cowan et al, in their study, also found that using educational videos yielded higher intravenous contrast knowledge scores than routine verbal informed consent procedures. 19 Migden et al found increased patient understanding (with patients scoring 91.6% on multiple choice quiz) in the video demonstration group as compared with a group who got only a live demonstration of wound care (84%). 20 These results are not in agreement with present study where live demonstration showed better knowledge retention as compared with video demonstration.

Studies by Luck et al found that patients who were given information before colonoscopy, by showing video demonstration along with written leaflets, showed less anxiety and better information retention than those who were just given instructions in the form of written leaflets. 21 These results are not in agreement with the present study where video demonstration and written leaflets provide no significant difference in knowledge retention levels.

Our study group patients were recalled after 1 week of delivering maintenance information, and the retention levels were evaluated. All three groups showed an increase in knowledge retention. The results were in accordance with the study by Fleischman and Garcia 22 who found an overall group retention rate of 26.5% after 20 minutes and 24.4% after 1 week, in patients undergoing surgery when instructions are given verbally and in written leaflets.

The question related to brushing of teeth showed improvement after the patient was educated. It improved from 90 to 100% in group 1, 85 to 95% in group 2, while in group 3, it remained the same at 95%. There was an increase in patient's motivation related to the number of times they brush their teeth. The number of patients brushing their teeth once daily decreased from 60 to 10% in group 1, 30 to 0% in group 2, and 30 to 10% in group 3. Most of the patients started brushing twice or thrice daily. Mechanical debridement is necessary for effective plaque control. Furthermore, there was a significant increase in the use of mouthwash by patients after they were given instructions. This drastically improved from 20 to 75% in group 1, followed by 30 to 65% in group 2, and 55% to 65 in group 3. There were changes in the type of mouth wash, cleaning tool, and type of toothpaste used by patients after instructions for at-home maintenance were given. There were differences between each group related to these changes. Group 1 showed maximum improvement in information retention related to cleaning of prosthesis (10–100%), followed by group 2 (5–85%), and group 3 showed the least increase (20–40%). Patients also showed improvement in retention of knowledge related to recall visits which varied in each group.

Agre et al 18 stated that delivering information to the patient by prerecorded videotapes is useful, as it ensures that the same consistent information is given to all patients. Also, video recordings can be repeatedly played, paused, and played again at will. These recordings can also be shared easily. On the contrary, we feel that live demonstrations can be more beneficial in disseminating information, as during live demonstrations, the doctor can ensure better patient attention and engage the patient to a better level. The patient even has an option to clarify doubts during live demonstrations which give an upper edge to live demonstrations as compared with prerecorded videos. Authors feel that if pre-recorded videos are long, it will be difficult for patients to focus. While giving live demonstrations, a checklist should be used to ensure that no information is missed.

To the best of our knowledge, there are no published studies comparing the modes of delivering at-home maintenance information after FPD treatment on the patient's understanding and the level of information retention. Thus, it is difficult to compare the present study results with other published research because of differences in conditions. Various other factors, like patient education level and socioeconomic status, can affect the patient understanding and knowledge retention. Additional studies are required to evaluate these. This study can guide dentists in selecting the appropriate mode of delivering maintenance information after FPD treatment.

## Conclusion

Within the limitations of this study, it can be concluded that the mode of delivering postoperative instructions after FPD treatment affects patient understanding level and information retention. Patients who were given live demonstrations showed the best results compared with video recordings and written leaflets.

### Clinical Implication

This study can guide dentists in selecting the appropriate mode of delivering post–maintenance information after FPD treatment.
